# Predisposing factors of long-term responsiveness in a cardio-metabolic cohort: Tehran Lipid and Glucose Study

**DOI:** 10.1186/s12874-021-01351-5

**Published:** 2021-08-09

**Authors:** Leila Cheraghi, Parisa Amiri, Golnaz Vahedi-Notash, Sara Jalali-Farahani, Davood Khalili, Fereidoun Azizi

**Affiliations:** 1 Research Center for Social Determinants of Health, Research Institute for Endocrine Sciences, Shahid Beheshti University of Medical Sciences, P.O.Box: 19395-4763, Tehran, Iran; 2 Department of Epidemiology and Biostatistics, Research Institute for Endocrine Sciences, Shahid Beheshti University of Medical Sciences, Tehran, Iran; 3 Prevention of Metabolic Disorders Research Center, Research Institute for Endocrine Sciences, Shahid Beheshti University of Medical Sciences, Tehran, Iran; 4 Endocrine Research Center, Research Institute for Endocrine Sciences, ShahidBeheshti University of Medical Sciences, Tehran, Iran

**Keywords:** Predisposing factors, Responsiveness, Cohort studies, TLGS

## Abstract

**Background:**

Non-participation in cohort studies, if associated with both the exposure and occurrence of the event, can introduce bias in the estimates of interest. This study aims to identify factors associated with follow-up participation in Tehran Lipid and Glucose Study, a large-scale community-based prospective study in West Asia.

**Methods:**

A sample of 10,368 adults from TLGS was included in the analysis. All analyses were split according to sex and age groups (20–39, 40–59, and 60 years). The associations between socio-demographic, health, and lifestyle factors with response rate were identified using the Generalized Estimating Equations model.

**Results:**

Over the median of 15.7 years of follow up the response rate was 64.5%. The highest response rate was observed in those aged 40–59 years for both sexes. Current smokers had lower odds of response in both sexes for all age groups, ranging from 0.51 to 0.74, *p* < 0.01. In young adults, being single (OR = 0.79, OR = 0.57, *p* ≤ 0.01, respectively for men and women) and unemployed (OR = 0.73, OR = 0.76, *p* ≤ 0.01, respectively for men and women) in both sexes, high physical activity in men (OR = 0.77, *p* < 0.01), high education (OR = 0.75, *p* = 0.02) and obesity (OR = 0.85, *p* = 0.05) in women were associated with lower response rate. For the middle-aged group, diabetes in men (OR = 0.77, *p* = 0.05) and hypertension (OR = 0.84, *p* = 0.05), and having a history of cancer (OR = 0.43, *p* = 0.03) in women were factors associated with lower response rates. Finally, interventions for both sexes (OR = 0.75, OR = 0.77, *p* ≤ 0.05, respectively for men and women) and being divorced/widow in women (OR = 0.77, *p* = 0.05) were the factors associated with the lower response rate in the elderly.

**Conclusions:**

Long-term participation was influenced by socio-demographic, health, and lifestyle factors in different sex- and age-specific patterns in TLGS. Recruitment strategies targeting these factors may improve participant follow-up in longitudinal studies.

**Supplementary Information:**

The online version contains supplementary material available at 10.1186/s12874-021-01351-5.

## Background

Non-communicable diseases (NCDs) – mainly cardiovascular disorders, cancers, chronic respiratory diseases, and type 2 diabetes – are the leading cause of death worldwide and are responsible for 73% of all global mortality [1]. Based on a World Health Organization (WHO) report in the Middle East and North Africa (MENA) regions, NCDs were responsible for 74 percent of all deaths in 2015 [2]. Existing evidence indicates that NCDs are among the significant health problems in Iran [3–5]. In Iran during 2019, 83.5% of all deaths and 78.1% of all burdens of diseases were due to NCDs [6]. Monitoring NCDs and related risk factors to prevent and control the burden of these diseases to promote population health in Iranian populations seems essential.

The cohort studies are the main research methods for understanding the etiology and prognosis of NCDs within populations by determining the causal effects of the environmental exposure factors that influence disease outbreaks. During the last decades, several valuable population-based cohort studies, including the Framingham [7], the Honolulu Heart [8], ARIC [9], CARDIA [10], and the CHS [11], have been established to investigate NCDs mainly in Western countries. To the best of our knowledge, Tehran Lipid and Glucose Study (TLGS) is the first large-scale community-based prospective study in West Asia focused on monitoring NCDs and related risk factors and lifestyle intervention to prevent these disorders in an urban population in Iran [12].

In cohort studies, selection bias due to non-participation or loss to follow-up represents a threat to the internal validity of results [13]. Evidence indicates that participation rates in cohort studies have decreased from 80% to 30–40% during the last two decades [14]. A large body of research revealed various reasons for non-participation in scientific studies, including increasing the number of studies, declining in volunteerism in the societies, participating in studies with an issue which is particularly salient to the participants’ lives, more complication in epidemiologic research involving survey assessments, biologic sampling, more complex consent procedures and frequently requests for ongoing follow-up [15]. In terms of cohort studies, the role of participants’ sex, age, marital status, working status, level of education, smoking, obesity, hypertension, and history of cardiovascular disease on their participation have been previously indicated [16–22]. These studies typically examined associated factors with the involvement in the Western population. Data available showed demographics, ethnicity, geographical and cultural differences between Eastern and Western countries, leading to a different pattern of NCDs and consequently participation in these regions [23]. Little information is available on the factors influencing participation in Eastern societies, particularly in West Asia [24].

To the best of our knowledge, this study is the first effort that clarifies factors associated with non-participation in the TLGS study as a population study in West Asia. Findings of the current study regarding the primary participants’ characteristics could underlie non-participation in a prospective cohort in West Asia add value to the previous findings that emerged from similar studies conducted in Western societies. Our results would be beneficial for identifying subgroups who are more likely to refuse participation in the studies and improve retention strategies to minimize this disruption.

## Methods

The protocol of the TLGS study was based on the WHO-recommended model for field surveys of diabetes and other non-communicable diseases and the WHO-MONICA protocol for population surveys [25, 26]. The design of this study encompasses two major components: phase 1 is a cross-sectional study for determining the prevalence of NCDs and their risk factors; implemented from 1999 to 2001, and phase 2 is a cohort and prospective interventional study, planned for the next 20 years. Primary, secondary and tertiary interventions were designed based on specific target groups, including schoolchildren, housewives, and high-risk persons. Officials of various sectors such as health, education, municipality, police, media, traders, and community leaders were actively engaged as decision-makers and collaborators. Interventional strategies were based on lifestyle modifications in diet, smoking, and physical activity through face-to-face education. A detailed description of the methodology, rationale, and design of the TLGS study has been previously published [12].

### Study population

A multistage stratified cluster random sampling technique was used to select people aged over three years from urban district 13 of Tehran, the capital of the Islamic Republic of Iran. Two important rationales for choosing district 13 were the high stability of the population residing in that district compared to the other districts of Tehran, and the age distribution of the population was representative of the overall population of Tehran at the beginning of the study [12]. All family members were invited in the cross-sectional phase 1 for baseline measurements and were followed every three years in ongoing prospective follow-ups. A total of 15,005 individuals aged ≥ 3 years, who agreed to participate in the study, were invited to the TLGS data-gathering center, and after signing informed written consent, studied by trained physicians according to the relevant protocol. Demographic, clinical, and laboratory data were collected for these participants. Lifestyle interventions were implemented on 5630 individuals in phase 2 of the TLGS, and 9375 individuals served as controls. For the current study, 10,368 adults aged ≥ 20 years were included in the analysis, of which 3931 individuals were in the intervention group, and 6437 people were in the control group. All participants signed written informed consent. The study was approved by the ethics committee of the Research Institute for Endocrine Sciences, Shahid Beheshti University of Medical Sciences.

### Anthropometric and clinical assessments

All participants were asked to fast for 12 h overnight and to avoid smoking and heavy exercise in the morning before coming to the Lipid and Glucose Research Unit. Medical history, clinical examination, demographic, and lifestyle information were obtained using a standard and validated questionnaire from invited participants. Based on the TLGS measurement protocol [12], weight was measured using a digital scale while wearing minimum clothing and no shoes; height was measured without shoes, standing position with shoulders in normal alignment. Waist circumference was measured at the umbilical level without any pressure to the body surface and was recorded to the nearest 1 cm. After a 15-min rest in the sitting position, two systolic and diastolic blood pressure measurements were taken on the right arm, using a standardized mercury sphygmomanometer (calibrated by the Iranian Institute of Standards and Industrial Researches). The mean of the two measurements was considered as the participant’s blood pressure. A blood sample was taken between 7:00 and 9:00 AM from all study participants, after 12 to 14 h of overnight fasting. All blood analyses were carried out at the TLGS research laboratory on the day of the blood collection. Details of laboratory measurements, including fasting blood glucose levels, 2 h plusma glucose, and serum creatinine, have been reported previously [12].

### Definition of terms

Baseline characteristics of participants, including age, sex, marital status, education level, occupation status, physical activity, smoking, general obesity, hypertension, diabetes, chronic kidney disease, history of cancer, cardiovascular disease, and intervention status, were considered in the present study.

Marital status was defined as single, married, and divorce or widow. Education level was considered as illiterate or primary, secondary, and higher. Occupation status was defined as employed and unemployed. Physical activity was categorized as low or moderate activity and high activity. Smoking was defined as smoker and non-smoker. General obesity was determined as non-obese (BMI < 30 kg/m^2^) and obese (BMI ≥ 30 kg/m^2^). Hypertension was defined as elevated blood pressure (≥ 140 mmHg systolic blood pressure or ≥ 90 mmHg diastolic blood pressure) or using antihypertensive medication. Diabetes was defined as fasting blood glucose ≥ 126 mg/dl or 2-h serum glucose ≥ 200 mg/dl or medical treatment. Chronic kidney disease (CKD) was defined as structural or functional kidney damage or GFR < 60 ml/min/1.73 m^2^ present for more than three months according to the Kidney Disease Outcome Quality Initiative (K/DOQI) guidelines [27]. History of cardiovascular disease included coronary heart disease (myocardial infarction, history of heart surgery, angioplasty, and hospitalization in the coronary care unit) and cerebrovascular attack.

### Response rate

The primary outcome of this study was response/participation rates during the follow-up periods, which were calculated as those participating in each follow-up examination divided by those eligible to participate in each follow-up. Those participants who experienced death before each follow-up period were not eligible for calculating the response rate and were removed from the denominator.

### Statistical analysis

All analyses were split according to sex and age groups. Participants' baseline characteristics were expressed as mean ± sd for continuous variables and frequency (percentages) for categorical variables. The number of participants, the cumulative number of dead people, the number of eligible participants, and response rates were calculated for each follow-up examination. Response rates during follow-up examination were displayed graphically for sex and age groups and intervention and control groups. The age groups were defined as 20–39, 40–59, and 60 years and older. The association of responding at each follow-up with socio-demographic, health, and lifestyle variables as well as with intervention status was analyzed in longitudinal structure for each sex and age group. Generalized Estimating Equations (GEE) models with a binomial distribution and logit link function were used, and the working correlation matrix structure was considered AR(1). In GEE models, participation at each follow-up, defined as a binary outcome (yes or no), was considered the dependent variable, and participants' baseline characteristics were considered independent variables. This model examined which individual baseline characteristics were associated with response rate during follow-ups. For each sex and age group, odds ratios (ORs) of the response and their 95% confidence intervals (CIs) were calculated for the exposure variables using two sets of univariate and multiple-adjustment models. Statistical analysis was done using IBM SPSS Statistics 22, and *p* < 0.05 was considered the significance level.

## Results

In the current study, a total of 10,368 adults (57.6% women) with a mean age of 42.75 ± 15.0 participated at baseline, and 3931 (37.9%) of them have been considered as the intervention group. The distribution of socio-demographic, health, and lifestyle characteristics of the study participants at baseline in total population and sex and age groups are presented in Table 1. Most participants at baseline were illiterate or had a primary level of education, and they were married and unemployed. However, in all age groups, employed and high-educated people mainly were men compared to women. Also, compared to women, the number of divorced or widowed male participants was less. Although the prevalence of smoking was increased in men compared to women in all age groups, the number of individuals who had moderate to low physical activity was similar for both men and women. Furthermore, for both sexes, the prevalence of chronic diseases was higher in older individuals. In this regard, the older women were more likely to have HTN, CKD, type 2 diabetes, history of cancer, and obesity than men.

**Table 1:** Baseline characteristics of study participants according to sex and age groups

**Variables**	**Total** (*n* = 10,368)	**Men** (*n* = 4395)			**Women** (*n* = 5973)		
**Age groups**		**20–39** (*n* = 1999)	**40–59** (*n* = 1468)	** ≥ 60** (*n* = 928)	**20–39** (*n* = 2963)	**40–59** (*n* = 2139)	** ≥ 60** (*n* = 871)
**Groups**							
Intervention	3931 (37.9)	721 (36.1)	541 (36.9)	388 (41.8)	1135 (38.3)	796 (37.2)	350 (40.2)
Control	6437 (62.1)	1278 (63.9)	927 (63.1)	540 (58.2)	1828 (61.7)	1343 (62.8)	521 (59.8)
**Education level**							
Illiterate or primary	5264 (50.8)	514(25.7)	664(45.3)	749(80.7)	896(30.2)	1601(75.1)	840(96.7)
Secondary	3829 (37.0)	1088(54.5)	518(35.4)	112(12.1)	1658(56.0)	430(20.2)	23(2.6)
Higher	1260 (12.2)	395(19.8)	283(19.3)	67(7.2)	708(13.8)	101(4.7)	6(0.7)
**Marital status**							
Married	8271 (79.8)	1236(61.8)	1447(98.6)	905(97.5)	2260(76.3)	1888(88.3)	535(61.4)
Single	1466 (14.1)	754(37.7)	12(0.8)	6(0.6)	658(22.2)	32(1.5)	4(0.5)
Divorced or widowed	631(6.1)	9(0.5)	9(0.6)	17(1.8)	45(1.5)	219(10.2)	332(38.1)
**Occupation status**							
Unemployed	6622 (63.9)	357(17.9)	250(17.0)	646(69.7)	2542(85.8)	1960(91.7)	867(99.5)
Employed	3738 (36.1)	1640(82.1)	1215(82.9)	281(30.3)	420(14.2)	178(8.3)	4(0.5)
**Current smoking**							
No	8806 (86.4)	1381 (70.8)	974 (67.6)	761 (83.3)	2835 (97.3)	2016 (95.5)	839 (97.8)
Yes	1381 (13.6)	570 (29.2)	466 (32.4)	153 (16.7)	78 (2.7)	95 (4.5)	19 (2.2)
**Physical activity**							
Low or moderate	7716 (75.9)	1505 (77.5)	1144 (79.6)	707 (77.3)	2162 (74.3)	1527 (72.5)	671 (78.5)
High	2450 (24.1)	438 (22.5)	293 (20.4)	208 (22.7)	748 (25.7)	579 (27.5)	184 (21.5)
**CKD**							
No	8167 (81.2)	1863(98.2)	1242(88.0)	563(61.7)	2753(95.4)	1438(68.5)	308(35.9)
Yes	1896 (18.8)	34(1.8)	170(12.0)	349(38.3)	132(4.6)	660(31.5)	551(64.1)
**Obesity**							
No	7779 (77)	1705 (87.3)	1206 (83.6)	773 (85.1)	2334 (81.7)	1213 (57.8)	548 (64.7)
Yes	2329 (23)	249 (12.7)	236 (16.4)	135 (14.9)	523 (18.3)	887 (42.2)	299 (35.3)
**Hypertension**							
No	7819 (77.1)	1783 (92.5)	1092 (76.2)	463(50.9)	2736(94.1)	1423(67.6)	322(37.6)
Yes	2320 (22.9)	144(7.5)	341(23.8)	447(49.1)	171(5.9)	683(32.4)	534(62.4)
**Diabetes**							
No	8585 (88.1)	1811(97.8)	1176(85.3)	663(73.0)	2727(97.7)	1686(82.2)	552(68.0)
Yes	1164 (11.9)	40(2.2)	203(14.7)	234(27.0)	63(2.3)	364(17.8)	260(32.0)
**CVD history**							
No	9844 (94.9)	1985(99.3)	1372(93.5)	765(82.4)	2947(99.5)	2038(95.3)	737(84.6)
Yes	524 (5.1)	14(0.7)	96(6.5)	163(17.6)	16(0.5)	101(4.7)	134(15.4)
**Cancer history**							
No	10,324 (99.6)	1999(100)	1461(99.5)	922(99.4)	2957(99.8)	2123(99.3)	862(99.0)
Yes	44 (0.4	0	7 (0.5)	6(0.6)	6(0.2)	16(0.7)	9(1.0)

Data were presented as frequency (%)

Table 2 shows the response rate and the number of people who participated in each follow-up examination in this cohort study. Of the 10,368 people who participated at baseline, 894 (9%) participants experienced death until the last examination. The cumulative number of dead people at each follow-up examination has also been presented in Table 2. These participants were not eligible for calculating response rates at each examination and therefore removed from the denominator. The response rate in the first follow-up examination was 61.5% (6232/10136), and were 66.6% (6654/9995), 68.3% (6701/9810), 65.6% (6319/9611) and 60.4% (5725/9474) in the subsequent examinations.

**Table 2:** The response rate of study participants in each follow-up examination

	**1**^**st**^** follow up examination**	**2**^**nd**^** follow up examination**	**3**^**rd**^** follow up examination**	**4**^**th**^** follow up examination**	**5**^**th**^** follow up examination**
Number of participants	6232	6654	6701	6319	5725
Cumulative number of individuals who experience death	232	373	558	757	894
Number of eligible participants	10,136	9995	9810	9611	9474
Response rate	61.5	66.6	68.3	65.6	60.4

Our results indicated that in male participants, individuals aged 40–59 or ≥ 60 years were more likely to be responsive than those aged 20–39 years (OR = 1.47, *p* < 0.001 and OR = 1.13, *p* = 0.010 respectively). In females, higher responsiveness was significant only in 40–59 years compared to other age groups (OR = 1.48, *p* < 0.001) (Table 3).

**Table 3:** The Odds ratio of responsiveness for age groups and follow-up examinations in males and females participants: The GEE model results

Variable	Male		Female	
	**Odds ratio** **(95% CI)**	***p*-value**	**Odds ratio** **(95% CI)**	***p*-value**
**Age groups**				
20–39	ref		ref	
40–59	1.46 (1.31–1.64)^a^	< 0.01^†^	1.48 (1.35–1.63)	< 0.01
≥ 60	1.44 (1.25–1.66)	< 0.01	0.92 (0.80–1.04)	0.19
**Examination**				
1^st^ examination	ref		ref	
2^nd^ examination	1.29 (1.21–1.38)	< 0.01	1.23 (1.17–1.29)	< 0.01
3^rd^ examination	1.48 (1.38–1.58)	< 0.01	1.29 (1.22–1.36)	< 0.01
4^th^ examination	1.31 (1.23–1.41)	< 0.01	1.15 (1.09–1.22)	< 0.01
5^th^ examination	1.03 (0.96–1.11)	0.34	0.93 (0.88–0.99)	0.01

^a^data are the Odds ratios and 95% confidence interval of responding in follow-up examinations

^†^*p-*values

**Fig. 1:**
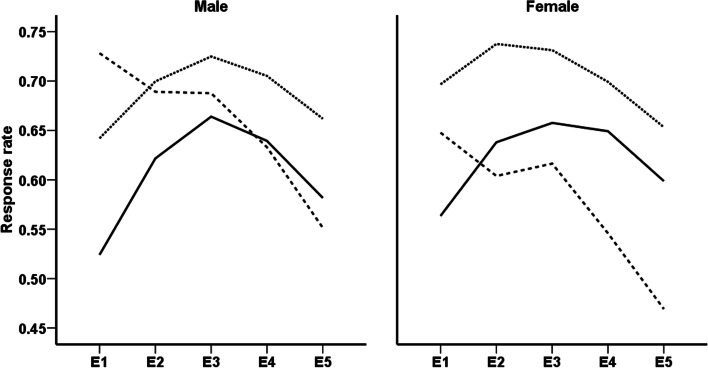
Sex-specific trends of response rates in different age groups; 20–39 years (solid line), 40–59 years (dotted line), and ≥ 60 years (dashed line). E1, E2, E3, E4, and E5 represent first to fifth follow up examinations, respectively

**Fig. 2:**
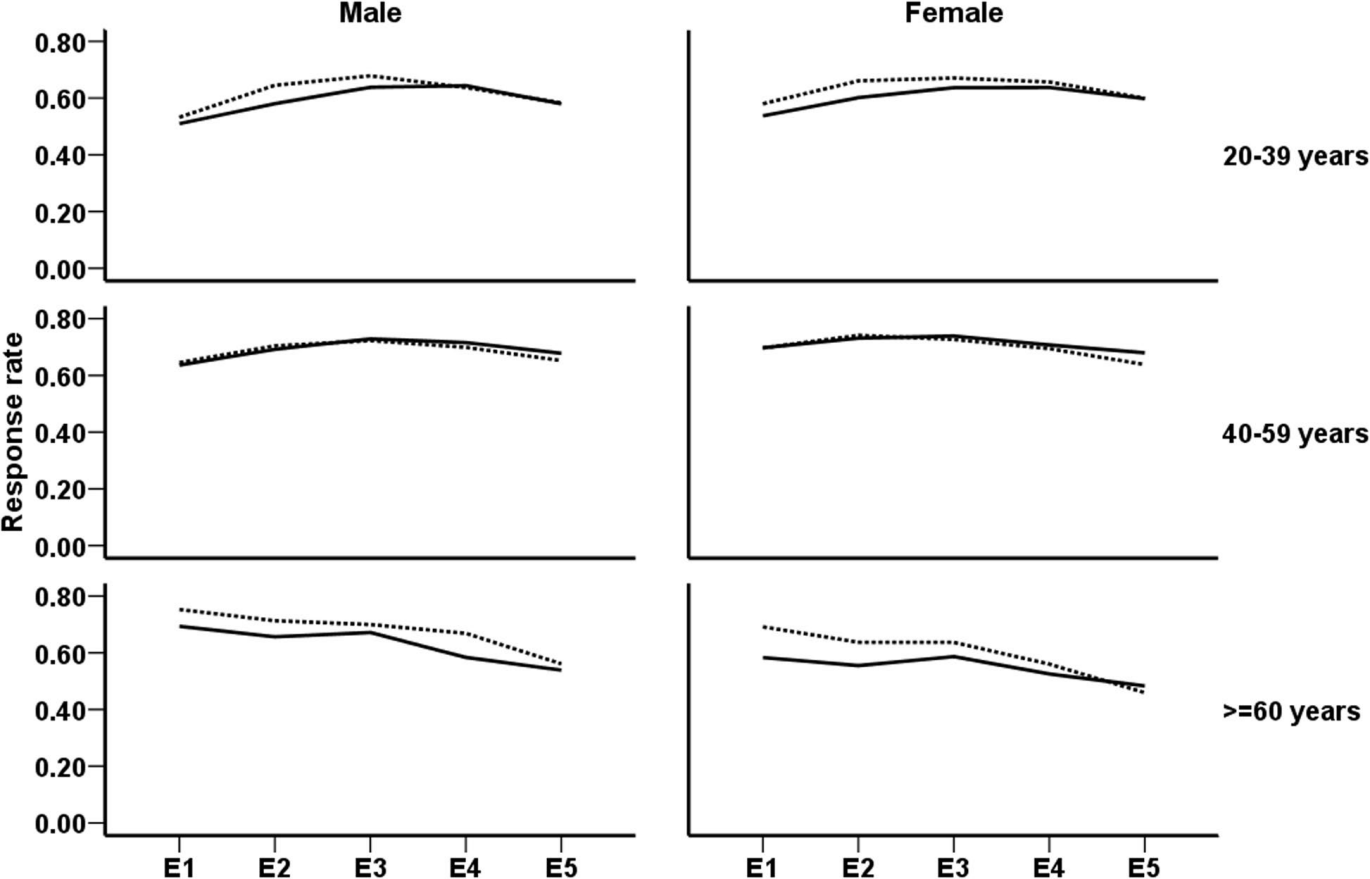
Sex- and age-specific trends of response rates in intervention and control groups; Solid line and dotted line represent response rate trend respectively in intervention and control groups. E1, E2, E3, E4, and E5 represent first to fifth follow up examinations, respectively

Comparison of response rates in men and women aged < 60 years showed an increasing trend in the initial follow-up examinations and a decreasing trend during the final assessments. The results also showed a reducing trend in response rates of older men and women during all follow-up examinations (Fig. 1). Figure 2 illustrated sex and age-specific response rates during a follow-up examination for intervention and control groups. According to the current results, the participation rates of men and women in the intervention and control groups were similar in those aged < 60 years. However, elderly participants in the intervention group had a lower participation rate than their counterparts in the control group.

The univariate results of GEE models regarding age and sex specific associations of socio-demographic, health and lifestyle variables, and intervention status at baseline with responding rates at each follow-up were presented in Appendix Table 1.

Table 4 illustrated the sex and age-specific multiple associations of intervention status and socio-demographic, health, and lifestyle variables with participants’ response rates at each follow-up. As results indicated, for 20–39 years, men, singles, current smokers, and individuals with high physical activity were less likely to participate in the follow-up examinations (OR = 0.79, *p* = 0.01, and OR = 0.74, *p* < 0.01 and OR = 0.77, *p* < 0.01, respectively). In addition, employed younger men were more likely to participate in the follow-up examinations (OR = 1.37, *p* = 0.006). The results for 20–39 years showed that high educated, singles, current smokers and women with obesity had a lower chance of responding in the follow-up examinations (OR = 0.75, *p* = 0.02, and OR = 0.57, *p* < 0.01 and OR = 0.54, *p* < 0.01, OR = 0.85, *p* = 0.05, respectively). In addition, compared to unemployed women in the 20–39 years age group, employed participants had significantly more odds of taking part in the follow-up examinations (OR = 1.32, *p* = 0.01).

**Table 4:** The results of multiple-adjusted GEE models in men and women

Variables	Men			Women		
**Age groups**	**20–39 years**	**40–59 years**	** ≥ 60 years**	**20–39 years**	**40–59 years**	** ≥ 60 years**
**Groups**						
Control	ref	ref	ref	ref	ref	ref
Intervention	0.87^a^(0.74–1.01) 0.06^†^	1.04 (0.86–1.26) 0.69	**0.75 (0.58–0.98)** **0.03**	0.92 (0.81–1.05) 0.21	1.06 (0.90–1.25) 0.47	**0.77 (0.60–1.00)** **0.05**
**Education level**						
Illiterate or primary	ref	ref	ref	ref	ref	ref
Secondary	1.01 (0.84–1.21) 0.92	0.89 (0.72–1.09) 0.24	1.20 (0.81–1.79) 0.37	0.91 (0.79–1.06) 0.24	0.92 (0.60–1.39) 0.69	0.73 (0.34–1.57) 0.42
Higher	0.91 (0.72–1.13) 0.38	0.84 (0.65–1.08) 0.18	1.38 (0.85–2.26) 0.20	**0.75 (0.60–0.95)** **0.02**	0.83 (0.67–1.02) 0.08	1.17 (0.35–3.90) 0.80
**Marital status**						
Married	ref	ref	ref	ref	ref	ref
Single	**0.79 (0.66–0.95)** **0.01**	0.59 (0.21–1.66) 0.31		**0.57 (0.49–0.66)** ** < 0.01**	1.04 (0.51–2.11) 0.91	
Divorced or widowed	0.92 (0.33–2.60) 0.88	1.39 (0.41–4.68) 0.60	1.33 (0.44–4.03) 0.62	1.13 (0.72–1.78) 0.58	0.82 (0.63–1.06) 0.13	0**.77 (0.59–1.00)** **0.05**
**Occupation status**						
Unemployed	ref	ref	ref	ref	ref	ref
Employed	**1.37 (1.09–1.71)** ** < 0.01**	0.97 (0.76–1.26) 0.85	1.08 (0.81–1.43) 0.59	**1.32 (1.07–1.63)** **0.01**	0.90 (0.65–1.25) 0.54	0.20 (0.03–1.35) 0.10
**Current smoking**						
No	ref	ref	ref	ref	ref	ref
Yes	**0.74 (0.63–0.88)** ** < 0.01**	**0.62 (0.51–0.76)** ** < 0.01**	**0.58 (0.41–0.83)** ** < 0.01**	**0.54 (0.37–0.79)** ** < 0.01**	**0.54 (0.38–0.76)** ** < 0.01**	0.66 (0.26–1.65) 0.37
**Physical activity**						
Low or moderate	ref	ref	ref	ref	ref	ref
High	**0.77 (0.64–0.91)** ** < 0.01**	0.92 (0.74–1.15) 0.47	0.99 (0.73–1.33) 0.93	1.07 (0.93–1.23) 0.38	1.00 (0.84–1.20) 0.98	1.35 (0.99–1.83) 0.06
**CKD**						
No	ref	ref	ref	ref	ref	ref
Yes	0.69 (0.38–1.25) 0.22	1.03 (0.77–1.38) 0.83	0.84 (0.64–1.09) 0.19	1.01 (0.74-1.37) 0.95	1.01 (0.85–1.21) 0.88	1.00 (0.77–1.31) 0.98
**Obesity**						
No	ref	ref	ref	ref	ref	ref
Yes	0.99 (0.78–1.25) 0.92	1.15 (0.90–1.48) 0.27	1.20 (0.83–1.73) 0.33	**0.85 (0.71–1.00)** **0.05**	0.91 (0.77–1.07) 0.24	1.11 (0.86–1.45) 0.42
**Hypertension**						
No	ref	ref	ref	ref	ref	ref
Yes	1.03 (0.76–1.38) 0.87	1.15 (0.92–1.45) 0.23	0.83 (0.63–1.10) 0.20	0.93 (0.70–1.23) 0.61	**0.84 (0.71–1.01)** **0.05**	1.03 (0.79–1.34) 0.85
**Diabetes**						
No	ref	ref	ref	ref	ref	ref
Yes	1.11 (0.69–1.78) 0.68	**0.77 (0.59–1.01)** **0.05**	0.91 (0.66–1.25) 0.56	1.23 (0.82–1.85) 0.32	0.92 (0.75–1.14) 0.50	0.83 (0.63–1.08) 0.16
**CVD history**						
No	ref	ref	ref	ref	ref	ref
Yes	0.60 (0.24–1.49) 0.27	0.83 (0.55–1.26) 0.39	0.73 (0.51–1.05) 0.09	1.18 (0.47–2.95) 0.73	0.75 (0.52–1.08) 0.12	0.94 (0.65–1.36) 0.73
**Cancer history**						
No		ref	ref		ref	ref
Yes		1.24 (0.48–3.15) 0.66	0.36 (0.08–1.64) 0.19		**0.43 (0.20–0.93)** **0.03**	4.26 (0.67–26.98) 0.12

^a^data are the Odds ratios and 95% confidence interval of responding in follow-up examinations. †*p-*values

The multiple adjusted results for 40–59 years men indicated lower odds of responding for current smokers and individuals with diabetes (OR = 0.62, *p* < 0.01 and OR = 0.77, *p* = 0.05 respectively). Furthermore, the results for women aged 40–59 years indicated that chance of response was lower in current smokers (OR = 0.54, *p* < 0.01), hypertensive women (OR = 0.84, *p* = 0.05), and in participants with a history of cancer (OR = 0.43, *p* = 0.03).

The results for older men illustrated that individuals in the intervention group were less likely to participate in the follow-up examinations than controls (OR = 0.75, *p* < 0.01). In addition, current smokers in this group had a lower chance of responding in the follow-up examinations (OR = 0.58, *p* < 0.01). The results for older women indicated decreasing responding chance in the intervention group (OR = 0.77, *p* = 0.05) and divorced or widowed individuals (OR = 0.77, = 0.05).

## Discussion

In the present study, the overall response rate during the median follow-up of 15.7 years was 64.5%, with the highest rate among middle-aged men and women. In general, declining response rate trends were observed for all age groups for both sexes during study follow-ups. In terms of predisposing factors, the current results indicated that among young participants, socio-demographic characteristics, including education, marital and employment status, as well as lifestyle behaviors, could determine response rates in a different sex-specific pattern. Weight status was the only cardiovascular risk factor influencing females’ response rate in this age group. However, among middle-aged participants, smoking and cardiovascular risk factors and history of chronic diseases were the most important factors, which could affect the response rates in a sex-specific manner. Finally, lifestyle behaviors and interventions and marital status were the most critical factors associated with responsiveness in the elderly.

Our results showed that in TLGS cohort study, the response rate in each follow-up was higher than 60% which has been accepted as a threshold of sufficient participation [28]. The current finding indicated a general decrease in response rate during the time. Declining in response rate also was observed in other cohorts such as the Framingham study [7] and the ARIC study [9]. Recruiting cohort subjects and carrying out follow-ups are becoming problematic in recent years [29]. In this study, age and sex differentials were observed, with a higher response rate among those aged 40–59 years for both sexes. In terms of youth and elderly, while young women were more responsive than the elderly, young men had lower response rates. Declining response rates in the elderly are expected due to deaths and disability attritions [30, 31]. The lower response rates among young male participants may relate to work obligations and family commitments and less time to participate in research [18]. The more inadequate response among younger males is consistent with reports indicating that younger age is associated with higher loss to follow-up, which may be explained by greater geographical mobility among younger subjects [32]. The young individuals may also perceive less disease susceptibility and less benefit from ongoing participation in long-term studies [19].

The findings of the current study indicated that in young adults, socio-demographic factors and lifestyle behaviors were the most significant determinants of response rates. To further clarify, being single, unemployed, and a smoker has been associated with lower response rates in young males and females. Current findings are in line with the results of previous studies, which indicated lower response rates in single participants compared to their married counterparts [33–36] and unemployed individuals compared to employed ones [36]. Higher response rates in married participants may be due to a more heightened sense of commitment than non-married counterparts and higher response rates in employed participants due to positive association between employment and contact and cooperation propensity [37]. In terms of lower response rates of smokers compared to non-smokers, in agreement with current findings number of previous studies indicated a distinct role of health-related behaviors in participation. In this regard, several studies showed lower response rates among smokers than non-smokers [36, 38]; moreover, another study reported smokers tend to respond late compared to non-smokers [33]. The possible reason lies in the cultural issues; smoking is not socially desirable behavior, and smoking by women is still a social taboo in Iran, making participants less inclined to talk about this issue. In addition, having higher levels of education and being obese in females, and having high levels of physical activity in male were significantly associated with lower response rates. In terms of the level of education, in contrary to current findings, previous studies reported lower response rates for those with low levels of education compared to their counterparts with higher educational attainments [33–35, 39, 40]; suggesting that less-educated individuals were more likely to refuse participation. This issue may be due to more appreciation of benefits of research activities by those with higher levels of education [41]. However, findings of study investigated determinants of non-response in health examination survey (HES) in Netherland indicated both very high and very low educated individuals had the lowest participation rates [42], which is consistent with findings of the current study. Similar to current results regarding lower response rates in obese women, other studies also reported that non-respondents had higher body mass index [38, 43]. Lower response rates in obese individuals may result from existing weight-related stigma and negative societal attitudes towards obese individuals in different settings [44]. Contrary to the findings of most previous studies, which indicated higher response rates in those with healthier lifestyle [45, 46], in the current study, lower response rates were observed for men with higher levels of physical activity. Physical activity in the current study includes both leisure time and occupational, physical activities; hence, men with more levels of physical activity may be those who are spending more time in occupational physical activity which is accompanied by expending more time and energy for job activities; therefore, busyness and fatigue caused by their occupational activities decrease possibility of participation of them in miscellaneous activities.

Based on the current findings, being a smoker and having a history of chronic diseases in both sexes of the middle aged were significantly associated with lower response rates. In addition, being a smoker only in men and being divorced/widow only in women and participating in intervention in both sexes were determinants of low response rates in the elderly. The findings of previous studies and possible reasons for more bass response rates in smokers have been addressed in the abovementioned paragraph. According to the current results, several previous studies indicated lower response rates in participants with chronic diseases regarding health status. For example, in ATTICA epidemiological study, those with diabetes and hypercholesteremia were found to have more missing data [45]. In another health survey in Finland, the probability of non-responding was higher among those with hypertension [33]. Lower response rates in participants with more inferior health status than healthy participants may be due to common physical and psychosocial problems associated with these diseases, which negatively influence chronically ill individuals' physical ability and motivation for participation. In addition, most of the individuals with chronic illness refer to the private section for treatment and receiving health care services; this may be another explanation for their motivation loss for participating in health surveys and, consequently, their lower response rates than their healthy counterparts. In line with the current findings, another study reported lower response rates in widowed and divorced individuals attributable to psychological distress following divorce and loss of their beloved one [33].

The strength of the present study is identifying subgroups that are most likely to be non-respondent during follow-up examinations in a cardio-metabolic cohort study conducted in the West-Asia region. In this regard, active recruitment strategies by paying particular attention to these subgroups may enhance cohort maintenance. In this regard, recruitment and retention strategies targeting these factors may improve participants’ commitment in longitudinal studies. Some limitations should be considered when interpreting our results. First, our findings from an urban-based survey regarding cardio-metabolic risk factors, which focused on one district of Tehran, may not generalize to cohorts with different socio-demographic characteristics or from other geographic regions**.** Second, the investigated potential determinants of the current response rate evaluation were based on baseline data, while some socio-demographic, health, and lifestyle factors might fluctuate or change over time. Finally, our results may not apply to clinical trials, requiring more frequent contact with trial subjects.

## Conclusions

Some socio-demographic, health, and lifestyle factors associated with follow-up participation in different sex- and age-specific patterns in TLGS were identified. These factors can be used to inform enrollment goals, subject recruitment methods, and novel targeted retention strategies for other longitudinal studies. Further investigations are required to assess the influence of the factors reported in our study over follow-up periods and the effectiveness of new retention strategies that incorporate these factors.

## Supplementary Information

**Additional file 1: Appendix table1.** The results of uni-variate GEE model in men and women.

## Data Availability

The datasets used and/or analysed during the current study are available from the corresponding author on reasonable request.

## Abbreviations

TLGS: Tehran Lipid and Glucose Study
NCDs: Non-communicable diseases
WHO: World Health Organization
BMI: Body Mass Index
CKD: Chronic kidney disease
K/DOQI: The Kidney Disease Outcome Quality Initiative
GEE: Generalized Estimating Equations
ORs: Odds ratios
CIs: Confidence intervals
HTN: Hypertension
ARIC: The Atherosclerosis Risk in Communities
