# Supplementary material for: Predisposing factors of long-term responsiveness in a cardio-metabolic cohort: Tehran Lipid and Glucose Study
Source: BMC Med Res Methodol. 2021 Aug 9;21:161. doi: 10.1186/s12874-021-01351-5 (PMC8353800; doi:10.1186/s12874-021-01351-5)
Supplement: Supplementary file 1 — Additional file 1: Appendix table1. The results of uni-variate GEE model in men and women. [file 12874_2021_1351_MOESM1_ESM.docx]

**Appendix table1.**The results of uni-variate GEE model in men and women

| **Variables** | **Men** | | | **Women** | | |
| --- | --- | --- | --- | --- | --- | --- |
| **Age groups** | **20-39 years** | **40-59 years** | **≥ 60 years** | **20-39 years** | **40-59 years** | **≥ 60 years** |
| **Groups** |  |  |  |  |  |  |
| Control | ref | ref | ref | ref | ref | ref |
| Intervention | 0.92^*^ (0.79-1.06)  0.24^†^ | 1.02 (0.85-1.22)  0.81 | 0.80 (0.62-1.02)  0.07 | 0.89 (0.79-1.01)  0.06 | 1.08 (0.92-1.26)  0.34 | **0.77 (0.61-0.98)**  **0.03** |
| **Education level** |  |  |  |  |  |  |
| Illiterate or primary | ref | ref | ref | ref | ref | ref |
| Secondary | 0.95 (0.81-1.12)  0.56 | 0.84 (0.70-1.03)  0.09 | 1.16 (0.80-1.70)  0.44 | **0.84 (0.73-0.96)**  **<0.01** | 0.87 (0.72-1.04)  0.13 | 0.63 (0.31-1.30)  0.21 |
| Higher | 0.96 (0.78-1.19)  0.72 | 0.86 (0.68-1.09)  0.20 | 1.42 (0.88-2.30)  0.15 | **0.77 (0.63-0.93)**  **<0.01** | 0.90 (0.64-1.28)  0.57 | 1.30 (0.39-4.28)  0.67 |
| **Marital status** |  |  |  |  |  |  |
| Married | ref | ref | ref | ref | ref | ref |
| Single | **0.74 (0.64-0.85)**  **<0.01** | 0.48 (0.20-1.17)  0.11 | 0.613 (0.07-3.36)  0.66 | **0.61 (0.53-0.70)**  **<0.01** | 1.07 (0.58-1.95)  0.83 | 0.81 (0.10-6.47)  0.84 |
| Divorced or widowed | 0.78 (0.28-2.18)  0.63 | 1.46 (0.43-4.91)  0.54 | 1.36 (0.51-3.65)  0.54 | 1.03 (0.65-1.63)  0.91 | **0.76 (0.60-0.97)**  **0.03** | **0.75 (0.59-0.96)**  **0.02** |
| **Occupation status** |  |  |  |  |  |  |
| Unemployed | ref | ref | ref | ref | ref | ref |
| Employed | **1.48 (1.24-1.77)**  **<0.01** | 0.95 (0.75-1.20)  0.66 | 1.11 (0.85-1.44)  0.45 | 1.06 (0.90-1.26)  0.49 | 0.84 (0.64-1.10)  0.20 | 0.15 (0.02-1.23)  0.08 |
| **Current smoking** |  |  |  |  |  |  |
| No | ref | ref | ref | ref | ref | ref |
| Yes | **0.78 (0.67-0.90)**  **<0.01** | **0.59 (0.49-0.70)**  **<0.01** | **0.56 (0.41-0.77)**  **<0.01** | **0.56 (0.39-0.80)**  **<0.01** | **0.54 (0.39-0.75)**  **<0.01** | 0.67 (0.29-1.50)  0.32 |
| **Physical activity** |  |  |  |  |  |  |
| Low or moderate | ref | ref | ref | ref | ref | ref |
| High | **0.72 (0.61-0.85)**  **<0.01** | 0.96 (0.78-1.18)  0.70 | 1.03 (0.78-1.37)  0.83 | 1.04 (0.91-1.20)  0.54 | 0.97 (0.82-1.15)  0.75 | **1.35 (1.01-1.80)**  **0.04** |
| **CKD** |  |  |  |  |  |  |
| No | ref | ref | ref | ref | ref | ref |
| Yes | 0.71 (0.41-1.26)  0.25 | 1.16 (0.88-1.53)  0.28 | 0.85 (0.66-1.10)  0.22 | 1.02 (0.77-1.37)  0.87 | 0.97 (0.83-1.15)  0.76 | 1.02 (0.80-1.31)  0.87 |
| **Obesity** |  |  |  |  |  |  |
| No | ref | ref | ref | ref | ref | ref |
| Yes | 1.05 (0.85-1.31)  0.63 | 1.22 (0.96-1.54)  0.11 | 1.20 (0.84-1.70)  0.32 | 0.97 (0.83-1.14)  0.75 | 0.89 (0.77-1.04)  0.15 | 1.09 (0.85-1.39)  0.50 |
| **Hypertension** |  |  |  |  |  |  |
| No | ref | ref | ref | ref | ref | ref |
| Yes | 1.05 (0.80-1.39)  0.71 | **1.24 (1.01-1.53)**  **0.04** | 0.88 (0.69-1.13)  0.33 | 0.91 (0.70-1.18)  0.46 | **0.84 (0.71-0.99**)  **0.03** | 1.04 (0.81-1.32)  0.78 |
| **Diabetes** |  |  |  |  |  |  |
| No | ref | ref | ref | ref | ref | ref |
| Yes | 1.28 (0.80 2.04)  0.30 | 0.83 (0.65-1.07)  0.15 | 0.91 (0.67-1.24)  0.55 | 1.30 (0.87-1.94)  0.20 | 0.89 (0.73-1.10)  0.28 | 0.79 (0.61-1.02)  0.07 |
| **CVD history** |  |  |  |  |  |  |
| No | ref | ref | ref | ref | ref | ref |
| Yes | 0.66 (0.30 1.67)  0.38 | 0.85 (0.58-1.24)  0.40 | **0.65 (0.47-0.90)**  **0.01** | 1.06 (0.49- 2.32)  0.876 | **0.70 (0.50-0.99)**  **0.04** | 0.89 (0.63-1.27)  0.52 |
| **Cancer history** |  |  |  |  |  |  |
| No |  | ref | ref |  | ref | ref |
| Yes |  | 1.69 (0.67-4.31)  0.27 | 0.39 (0.09-1.70)  0.21 |  | **0.41 (0.19-0.91)**  **0.03** | 2.15 (0.47-9.74)  0.32 |

*data are the Odds ratios and 95% confidence interval of responding in follow-up examinations.†p-values
